# A child with duchenne muscular dystrophy: A case report of a rare diagnosis among Africans

**DOI:** 10.1002/ccr3.3254

**Published:** 2020-08-18

**Authors:** David D. Nassoro, Liset Torres, Rehema Marando, Lazaro Mboma, Seraphine Mushi, Issakwisa Habakkuk Mwakyula

**Affiliations:** ^1^ Department of Internal Medicine Mbeya Zonal Referral Hospital Mbeya Tanzania; ^2^ Department of Internal Medicine The University of Dar es Salaam Mbeya College of Health and Allied Sciences Mbeya Tanzania; ^3^ Department of Pathology Mbeya Zonal Referral Hospital Mbeya Tanzania; ^4^ Department of Pathology The University of Dar es Salaam Mbeya College of Health and Allied Sciences Mbeya Tanzania; ^5^ Department of Pediatrics and Child Health Mbeya Zonal Referral Hospital Mbeya Tanzania; ^6^ Department of Pediatrics and Child Health The University of Dar es Salaam Mbeya College of Health and Allied Sciences Mbeya Tanzania; ^7^ Department of Surgery Mbeya Zonal Referral Hospital Mbeya Tanzania; ^8^ Department of Surgery The University of Dar es Salaam Mbeya College of Health and Allied Sciences Mbeya Tanzania; ^9^ Department of Physiotherapy Mbeya Zonal Referral Hospital Mbeya Tanzania

**Keywords:** genetics, neurology

## Abstract

In Africa, lack of awareness and low index of suspicion of rare diseases like dystrophinopathies, directly or indirectly, contributes to the increased morbidity and mortality. Therefore, even though the data on prevalence is limited, we need to have a high degree of suspicion in patients presenting with suggestive clinical features.

## Graphical abstract

In Africa, lack of awareness and low index of suspicion of rare diseases like dystrophinopathies, directly or indirectly, contributes to the increased morbidity and mortality. The picture is a muscle biopsy of an 11‐year‐old boy with Duchenne muscular dystrophy showing a transverse section with degenerated skeletal muscles of variable size and necrotic background.

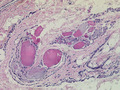

## 1 INTRODUCTION

Case reports of Duchene muscular dystrophy in Africa are rare. This may be because of the paucity of knowledge of dystrophinopathies. Therefore, the overall expected outcome in these patients is poor. We present a case of an 11‐year‐old boy with clinical features of dystrophinopathy.

Duchenne muscular dystrophy (DMD) is an early‐onset, severe, rapidly progressive neuromuscular disease belonging to a pathological group of diseases known as dystrophinopathies with muscle weakness as the primary clinical manifestation.^1^, ^2^

DMD is a debilitating early‐onset disorder associated with a functional deficiency of dystrophin. The affected individuals are wheelchair‐bound by the age of twelve and often succumb to failure of either cardiac or respiratory or both by the mid to late twenties. A milder form of the disease, Becker muscular dystrophy (BMD), is distinguished from DMD by delayed onset, later dependence on wheelchair support, and longer life span.

DMD occurs due to mutation in the X‐linked recessive gene coding the dystrophin protein.^3^ Globally, 1 in every 3500 male births is affected by DMD with regional variation of 0.95 and 16.76 per 100,000 observed in some areas of sub‐Saharan Africa and European countries, respectively.^4^, ^5^, ^6^

In developing countries, DMD is associated with early mortality, usually in the late teens or during the early third decade.^7^ Diagnosis of muscular dystrophies requires a comprehensive medical history, noting the distribution of weakness, age of onset, family history, and disease‐specific features. In many African countries, a combination of poor healthcare, illiteracy, late diagnosis, and delayed treatment may lead to increased morbidity and mortality.^8^

Moreover, in Africa, there is a paucity of DMD epidemiological studies, let alone case reports.^8^ The shortage of literature may be due to multiple factors, including lack of consideration of DMD as either provisional or differential diagnosis. In a few articles that studied the prevalence of DMD using genetic testing, the prevalence among blacks in South Africa was as low as 1/4, 000.^2^ These reported findings suggested unique mutations that were not detected by readily available genetic diagnostic tests. Furthermore, diagnostic errors cause underreporting, which is common even in tertiary hospitals and has been reported to decrease diagnostic yield.^2^, ^9^

## 2 CASE PRESENTATION

An 11‐year‐old male patient reported to the medical clinic with a history of generalized weakness for more than six years. Over the years, he received treatment in several hospitals where the definitive diagnosis was not reached. He received several empirical treatments like multivitamins and antibiotics, with minimal to no clinical improvement.

The parent reports a history of poor performance in school, repeated falls, inability to keep up with peers during sports due to excessive fatigue, progressive muscle weakness, and inability to climb stairs. There was no history of muscle pain, preceding trauma, or clinical features suggestive of an infection. Family history revealed that his maternal uncle, currently in his late teens, has experienced similar symptoms and is now bedridden.

**Figure 1:**
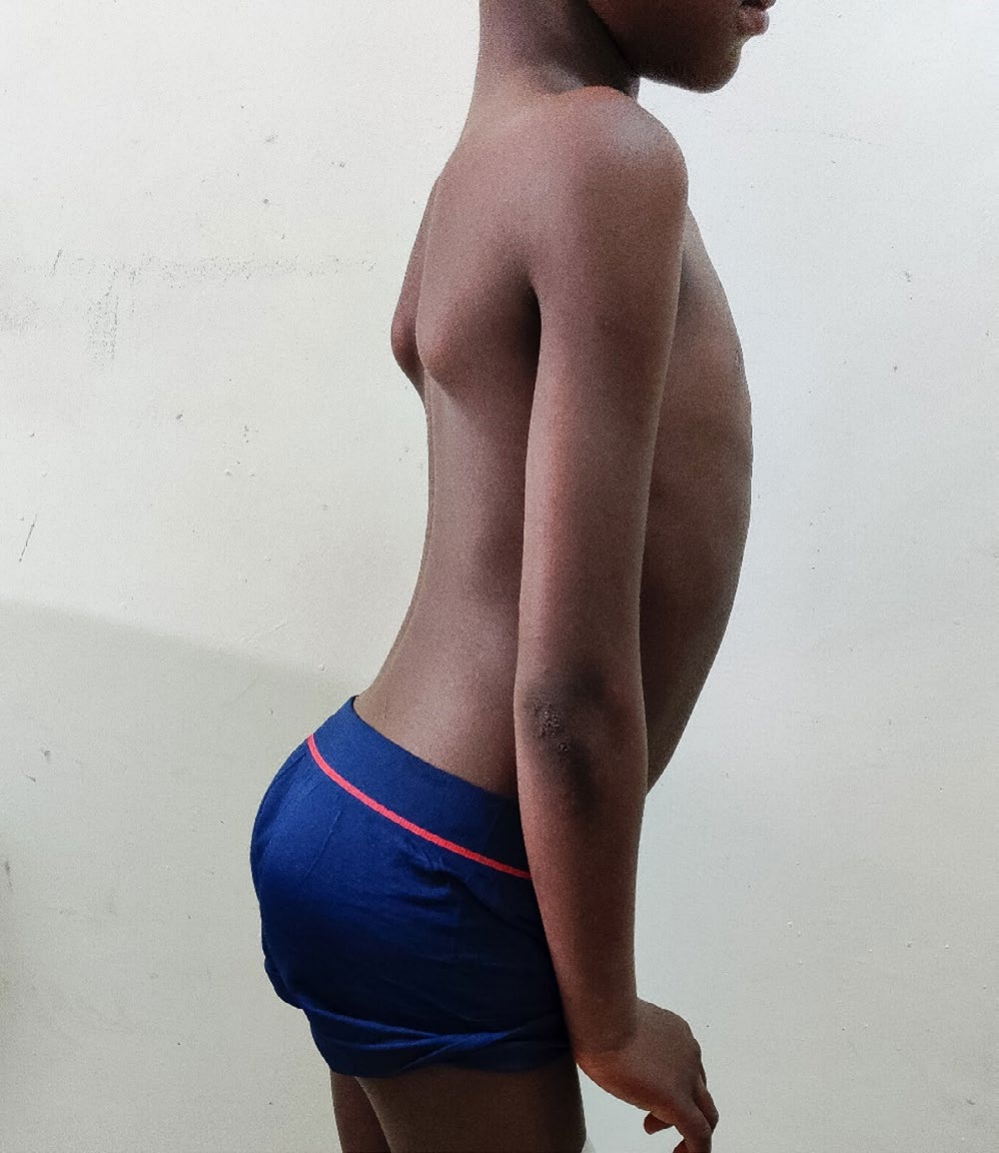
Lumbar Hyperlordosis

**Figure 2:**
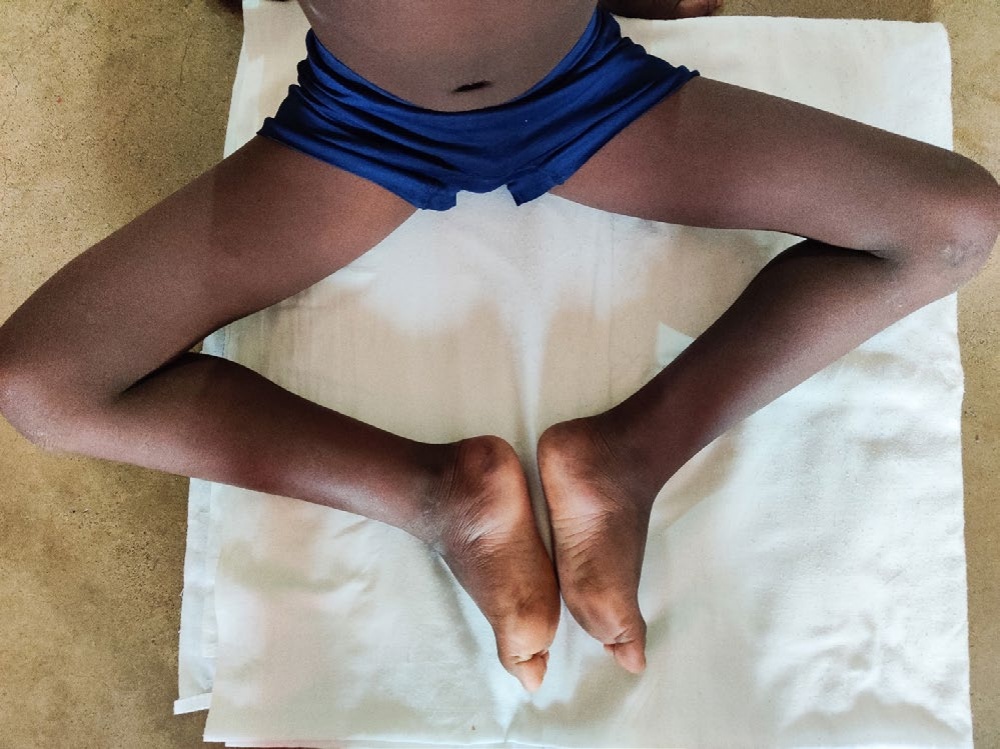
Flat feet (Pes Planus)

**Figure 3:**
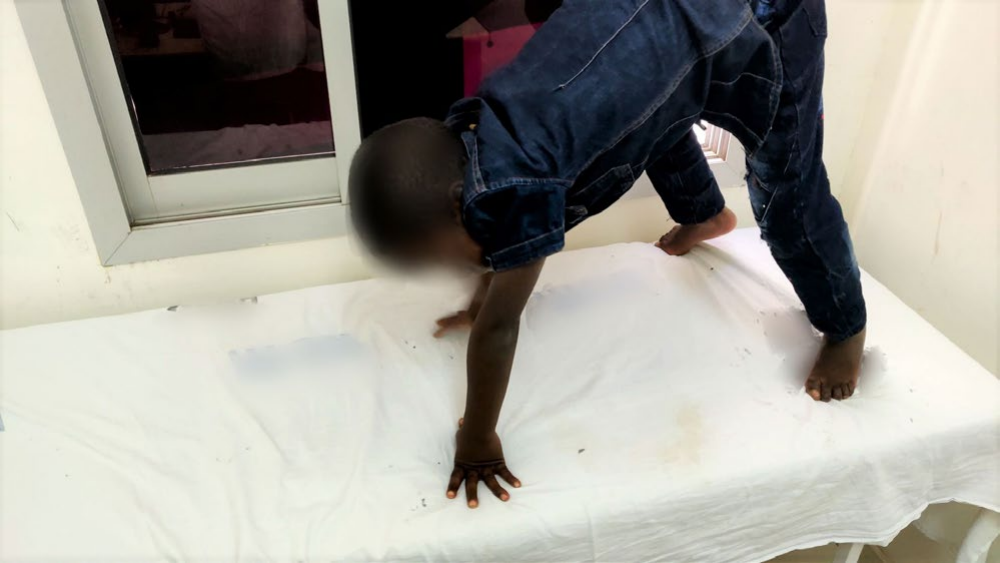
Part of Gowers’ sign

On examination, the child has slight wasting, fully conscious with a Montreal cognitive assessment score of 20, which indicates significant impairment of cognitive function. Furthermore, he was afebrile (36.7°C), and exhibits a waddling gait, lumbar hyperlordosis, toe walking, reduced power of proximal muscles of the lower limbs, calf hypertrophy, flat feet (pes planus), and positive Gowers’ sign. Cranial nerve examination was unremarkable, and there were no apparent signs of either upper or lower motor lesions (Figures 1, 2, 3).

**Figure 4:**
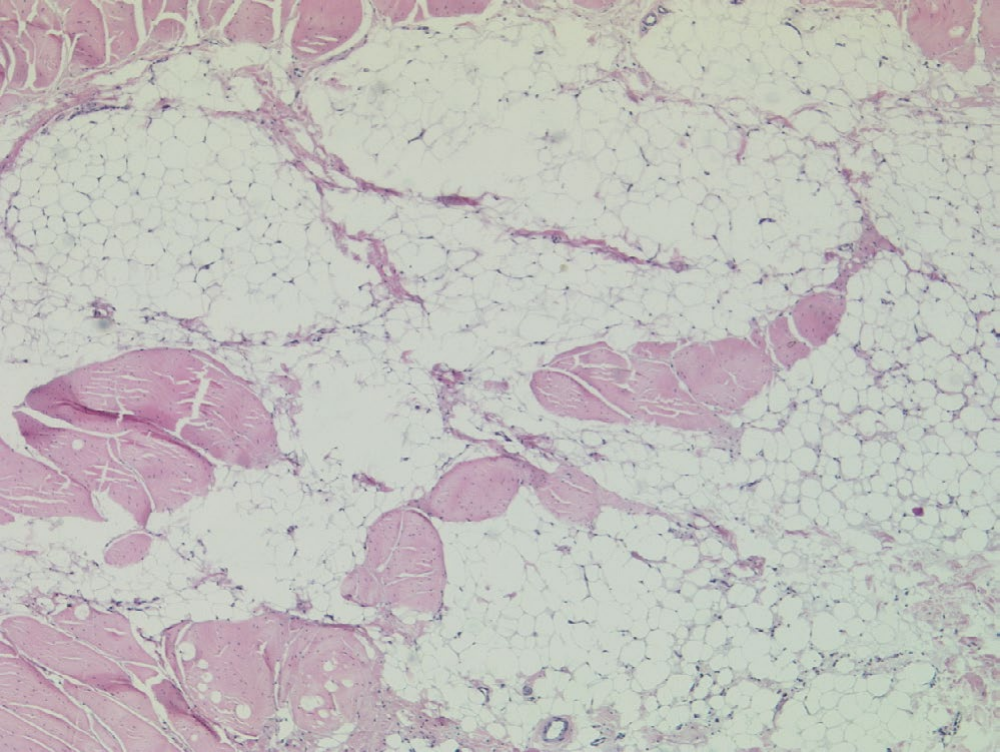
Longitudinal section showing extensive areas of atrophic skeletal muscle fibers replaced by fat

**Figure 5:**
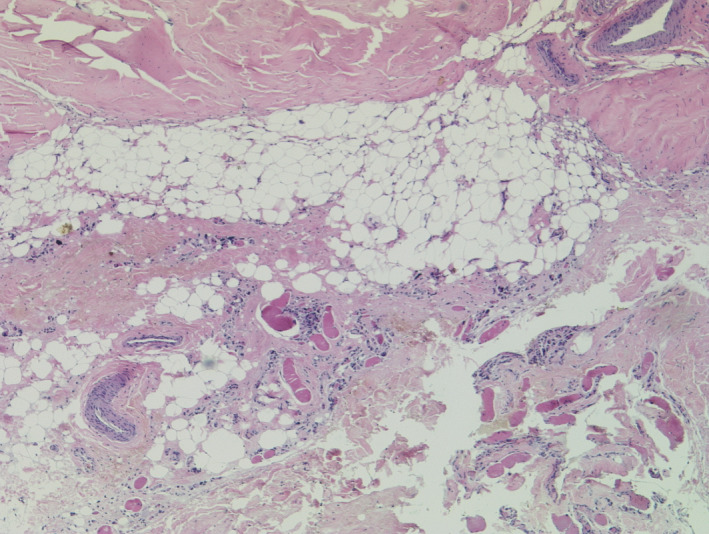
Longitudinal section showing skeletal muscle fibers of variable size replaced by fat tissue and fibrosis.

**Figure 6:**
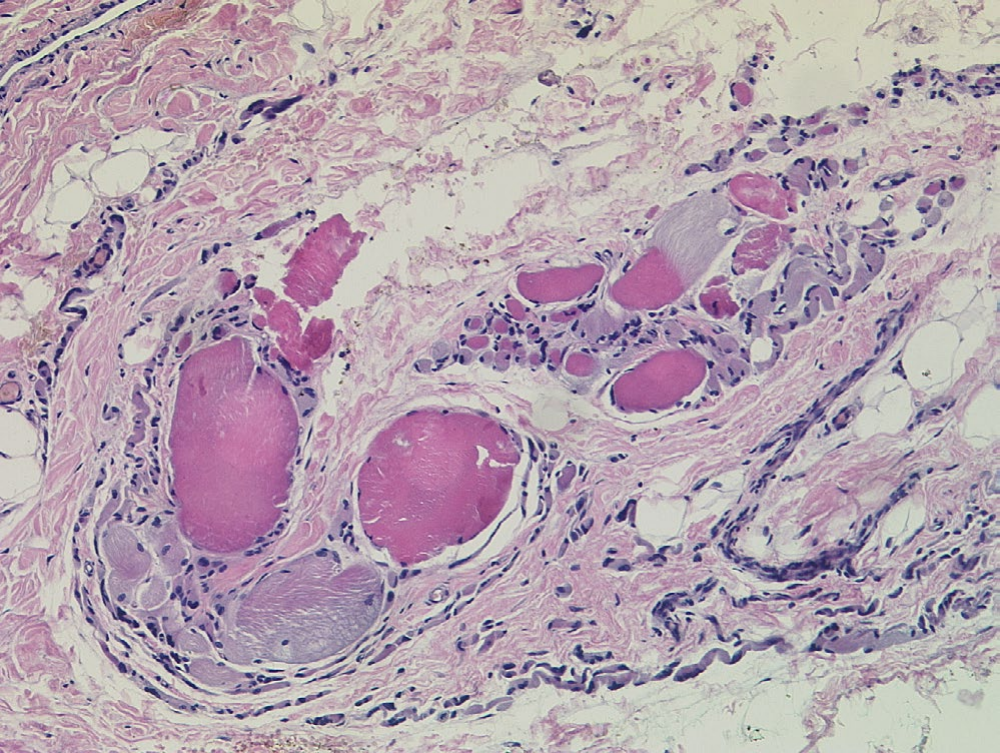
Transverse section with degenerated skeletal muscles of variable size and necrotic background.

We performed various laboratory investigations. While complete blood count was uneventful, biochemistry analysis showed significantly elevated levels of creatine kinase, alanine aminotransferase, aspartate aminotransferase, and lactate dehydrogenase (20 232.3 U/L, 206 U/L, 192.4 U/L and 645 µg/dl, respectively). Muscle biopsy results revealed an extensive loss of skeletal muscle fibers that are replaced by fat tissue, and extensive fibrosis noted. Echocardiogram and electrocardiogram were essentially normal. Therefore, based on history, clinical examination, and investigations, the diagnosis of DMD was established. (Figures 4, 5, 6).

The child was referred to a pediatrician who oversees his treatment and general well‐being, including regular assessment of respiratory and cardiac complications. Furthermore, he is on systemic glucocorticoids and also attends regular physiotherapy sessions.

## 3 DISCUSSION

### 3.1 The molecular aspect of dystrophinopathies

Duchenne muscular dystrophy and Becker muscular dystrophy share similar pathophysiology as the clinical manifestation is primarily due to a decrease in the production of dystrophin, which is a protein that primarily maintains the integrity of muscle fiber and interstitium.^10^ The gene is located at chromosome Xp21.2 and spans approximately 2.3 megabases, making it the largest gene identified in humans. The protein product after transcription is dystrophin, and it weighs 427 kilodaltons (kD).^11^

The primary pathology is the deletion of exons in more than 60% of patients with DMD and BMD.^12^, ^13^ Partial gene replication is also evident in a minority of patients.^13^

Dystrophin is located in the cytoplasmic part of the muscle fibers, providing mechanical reinforcement to the sarcolemma and stabilizes the glycoprotein complex, shielding it from degradation.^10^ The loss of these membrane proteins usually initiates muscle fiber degeneration that eventually results in muscle weakness.

Dystrophin has several functional subunits, including dystrophin‐associated proteins such as neuronal nitric oxide synthase (nNOS), which produces nitric oxide that is important in increasing blood flow to the muscle. Therefore, this is a critical component in the prevention of early muscle fatigue, especially during exercise.^14^, ^15^, ^16^

The reduction of nitric oxide due to the impaired function of nNOS may create oxidative stress within myocytes. This process will eventually increase the production of inducible nitric oxide, which binds and destabilizes the ryanodine receptor in the sarcoplasmic reticulum, resulting in an overall increase in intracellular calcium. ^17^, ^18^, ^19^ This cascade of events results in the activation of calpans and eventually leads to the activation of muscle proteolysis.^20^

### 3.2 Clinical features

Weakness is one of the most characteristic clinical manifestations of DMD with proximal lower limb and truncal weakness affected first, then upper limb, and distal muscles following later. The age of onset is usually under five, and the affected child presents with difficulty in running, jumping, and walking up the stairs. On physical examination, waddling gait, calf enlargement, and lumbar lordosis are a common observation. These clinical features are in line with the presentation of our patient. Furthermore, clinical manifestations that lead to weakness, such as Gowers' sign, have been shown to have lower sensitivity and high specificity to DMD.^21^ However, clinical features such as age at onset and rapidity of disease progression, narrow the diagnosis further, and increase suspicion. Our patient's age of onset and progression of the disease are in line with the classical presentation of DMD. Other motor signs and symptoms include decreased endurance, flat feet, frequent falls, muscle pain with or without cramping, and toe walking. Patients are usually wheelchair‐bound by the age of twelve.^22^

Orthopedic complications usually result from progressive weakening of truncal muscles that eventually lead to neuromuscular scoliosis after the loss of ambulation.^23^, ^24^ However, steroids and wheelchair fitting may delay this clinical presentation. If scoliosis is present by the time of presentation, surgical correction by posterior fusion can improve the general condition of the patient by improving the stability when sitting on a wheelchair, respiratory function, and reduction of risk of pressure sores.^25^ The data on posterior fusion is mostly retrospective; however, clinical experience has shown beneficial effects in improving the overall quality of life in this patient population. Early intervention advised, especially when the spine is still stable and straight.^25^ Decrease bone density, significant muscle weakness, and diminished power increase the risk of bone fractures. Furthermore, glucocorticoids, especially as disease‐modifying agents, have been shown to increase the risk of vertebrae fractures.^26^

Progressive respiratory system manifestations tend to increase from the time the patient starts using a wheelchair. They include ventilatory insufficiency, sleep‐disorder breathing, increased frequency of respiratory tract infection, and diminished cough capacity.^27^ Ventilatory insufficiency resulting from respiratory muscle weakness is implicated as one of the leading causes of morbidity and mortality.^28^ With the progression of scoliosis, the lung volume decreases progressively, leading to restrictive lung disease.^29^ Due to reduced cough reflex, these patients have been experiencing upper respiratory tract infections and frequent severe lower respiratory failure due to pneumonia. Routine cough training is generally essential as it improves their ability to clear airways and reduce the incidence of atelectasis.^30^ Expiratory muscles are generally more affected than Inspiratory muscles in DMD.^31^ Obstructive sleep apnea (OSA) is one of the most common clinical manifestations in DMD patients with advanced disease. Other associated complications are hypertension, stroke, and other cardiovascular comorbidities, to mention a few.^31^, ^32^ Patients with suggestive respiratory manifestations are supposed to undergo polysomnography for definitive diagnosis.

Like BMD, cardiac involvement is inevitable as dystrophin is also present in cardiac myocytes.^33^ There was a heterogeneous pathological alteration of ventricular myocardium that was said to be a result of combined consequences of myocardial wasting and remodeling due to decreased systolic function secondary to progressive cardiac muscle destruction.^34^, ^35^ The latter process occurs in combination with fibrosis and secondary fatty infiltration upon myocyte death. The incidence of cardiomyopathy in DMD patients increases gradually during the second decade of life. One study showed that cardiomyopathy was observed in about one‐third of patients by the age of 14, one half by 18 years, and all patients older than 18years.^36^ Despite the high incidence of dilated cardiomyopathy in DMD patients, most of them are relatively asymptomatic until late in the disease course; this has been explained by the inability of patients to exercise as the disease progresses hence masking the functional impairment that is generally one of the first markers of heart failure. Conduction abnormalities such as supraventricular dysrhythmias are also common in this patient population.^37^

Dystrophin has been reported in tissues other than muscle, such as in the retina, kidneys, and central nervous system.^38^ Expectedly, DMD patients experience an increased incidence of neuropsychological, neurobehavioral, and cognitive impairment with the progression of the disease.^39^ Relative to the general population, DMD was also associated with increased rates of obsessive‐compulsive disorder, anxiety, and attention deficit disorder.^40^, ^41^

Serum creatine kinase (CK) almost always significantly elevated in patients with DMD, and it mostly peaks at the age of two years. In some instances, the value can be 20 times higher than the upper limit of normal. However, this value decreases progressively as more muscle cells are replaced by fatty cells and fibrous tissue.^42^ Other enzymes such as aldolase, alanine transaminase, aspartate transaminase, and lactate dehydrogenase may be elevated.

The definitive diagnosis of DMD is reached after a combination of clinical features such as delayed milestones, Gowers' sign, toe walking, calf hypertrophy, laboratory tests such as creatine kinase, genetic testing, and sometimes muscle biopsy, especially if genetic tests are inconclusive. One study done in Pakistan by Hashim et al. (2011) showed that in cases of DMD, creatine kinase had a sensitivity and negative predictive value of 100% and specificity and positive predictive values of 91% and 88.8%, respectively.^1^ In Africans, some studies have shown that genetic tests came back positive in only up to 22% of biopsy‐confirmed, indicating there may be a high prevalence of unique mutations in this population that impair the genetic diagnosis of DMD.^2^ This finding suggests that in Africans, biopsy has higher sensitivity and specificity, reaching a definitive diagnosis compared to genetic testing. However, recent advances in genetic testing have significantly increased sensitivity even though the cost is quite prohibitive.^43^

Currently, there is no definitive treatment of DMD. Most patients die before reaching the third decade of life without appropriate medical care, most commonly from respiratory insufficiency and cardiac complications such as arrhythmias and cardiomyopathy. However, glucocorticoids have been the mainstay of treatment and improve, albeit modestly, motor, and pulmonary functions. Furthermore, they have an added benefit of reducing the risk of development of scoliosis, delaying cardiac complications, and even loss of ambulation.^44^ The effects of novel drugs such as Eteplirsen and Ataluren mainly depends on the genetic mutation involved in DMD. Clinical trials that evaluated those treatment options have shown the drugs to be quite useful in decreasing morbidity and mortality; however, even if the mutation warrants their use, the costs of these drugs have been reported to be quite prohibitive, reaching at least USD 300,000/= per year.^45^ Other reported supportive treatment options include physiotherapy sessions, managing complications such as heart failure and respiratory complications due to muscle weakness, and vertebrae complications.

### 3.3 Genetic Diagnosis of DMD in sub‐Saharan Africa

In most of sub‐Saharan Africa, the regular availability of genetic tests to diagnose dystrophinopathies is still a challenge. Even if they are available, the price tends to be prohibitive. Therefore, a biopsy is still the most logical investigation to diagnose DMD and other dystrophinopathies.

The use of the multiplex polymerase chain reaction (mPCR) was one of the most reliable genetic diagnostic test dystrophinopathies until when multiplex ligation‐dependent probe amplification (MLPA) was described in 2007.^2^, ^3^ However, since mPCR consists of multiple primer sets within a single primer mixture, it detects predefined mutations, and that is generally one of its greatest weakness.^4^ Furthermore, it may explain the observed low sensitivity in earlier studies done in Africa.^5^ Adopting these genetic tests has been slow due to their prohibitive cost, particularly to many low‐income African countries.^6^

There are few studies assessed the genetic characteristics of dystrophinopathies mutations in Africans. One article that studied mutation characteristics in South Africans Dystrophinopathies patients found that black patients had significantly more distal mutations as compared to whites and Asians.^46^ Moreover, in the study, MLPA had considerable sensitivity, albeit the sample size was relatively small and, therefore, not entirely representative of the general population. Also, biopsy is generally the most preferred diagnostic test in developing countries, expectedly, because of the prohibitive cost of genetic tests. However, more extensive genetic studies that assess the unique mutations in the patient population are needed, even though the price of performing those tests is still high.^47^

### 3.4 Differential diagnoses

It is essential to rule out other dystrophinopathies when considering DMD diagnosis.^48^ Limb‐Girdle muscular dystrophy (LGMD) is another differential diagnosis to DMD and can present strikingly similar to DMD; however, clinical manifestations usually tend to be apparent during the second decade, cognitive function is generally normal, calf pseudohypertrophy is often not marked, and levels of transaminases are usually not significantly elevated. Emery‐Dreifuss muscular dystrophy is another differential to DMD and presents with contractures, humeroperoneal muscle weakness, wasting, and cardiac manifestations such as arrhythmia and cardiomyopathy.^47^, ^48^ Another differential is Spinal Muscular Atrophy, whose highlighting feature is the various manifestation of lower motor neuron lesion.

## 4 CONCLUSION

When clinical features of dystrophinopathies are present, it is logical to think of DMD as the primary etiology as it is the most common cause. However, it is also essential to keep in mind other diagnoses that can present similarly to DMD. BMD is usually considered the milder form of the disease. The clinical features tend to manifest relatively late, with the initial manifestation commonly being heart failure during the second decade. Although the prevalence of DMD is perceived to be low in Africans, few studies are available to validate this assumption. Older genetic tests have reduced sensitivity, and newer tests are quite prohibitive and rarely available. Therefore, it is essential to conduct observational studies such as case‐control that may help us determine the unique genetic mutations in Africans that will improve the diagnostic accuracy and reduce the dependence of biopsy. Furthermore, clinicians should increase the degree of suspicion for DMD and other dystrophinopathies in any patient and especially children and teenagers, presenting with an early‐onset progressive weakness associated with clinical features suggestive of dystrophinopathies such as waddling gait, toe walking, and Gowers' sign.

## CONFLICT OF INTEREST

None declared.

## AUTHOR CONTRIBUTIONS

DN: contributed to the literature review and wrote the manuscript. SM: initiated the case‐report. LT: analyzed tissue biopsy, DN and RM were the primary clinicians involved in the patient's assessment and management. IM: was the clinical supervisor of this case. All authors: corrected and approved the final form of the manuscript.

## CONSENT FOR PUBLICATION

The patient parent gave written informed consent for publication of this case report.
